# Blood neurofilament light levels segregate treatment effects in multiple sclerosis

**DOI:** 10.1212/WNL.0000000000009097

**Published:** 2020-03-17

**Authors:** Bénédicte Delcoigne, Ali Manouchehrinia, Christian Barro, Pascal Benkert, Zuzanna Michalak, Ludwig Kappos, David Leppert, Jon A. Tsai, Tatiana Plavina, Bernd C. Kieseier, Jan Lycke, Lars Alfredsson, Ingrid Kockum, Jens Kuhle, Tomas Olsson, Fredrik Piehl

**Affiliations:** From the Department of Medicine Solna, Clinical Epidemiology Division (B.D.), The Karolinska Neuroimmunology & Multiple Sclerosis Centre, Department of Clinical Neuroscience (A.M., I.K., T.O., F.P.), and Institute of Environmental Medicine (L.A.), Karolinska Institutet; Centre for Molecular Medicine (A.M., I.K., T.O., F.P.), Karolinska University Hospital, Stockholm, Sweden; Neurologic Clinic and Policlinic, Departments of Medicine, Biomedicine, and Clinical Research (C.B., Z.M., L.K., D.L., J.K.), and Clinical Trial Unit, Department of Clinical Research (P.B.), University Hospital Basel, University of Basel, Switzerland; Sanofi Genzyme (J.A.T.), Stockholm, Sweden; Biogen (T.P., B.C.K.), Cambridge, MA; Department of Neurology, Medical Faculty (B.C.K.), Heinrich-Heine University, Duesseldorf, Germany; and Institution of Neuroscience and Physiology (J.L.), Sahlgrenska Academy, University of Gothenburg, Sweden.

## Abstract

**Objective:**

To determine factors (including the role of specific disease modulatory treatments [DMTs]) associated with (1) baseline, (2) on-treatment, and (3) change (from treatment start to on-treatment assessment) in plasma neurofilament light chain (pNfL) concentrations in relapsing-remitting multiple sclerosis (RRMS).

**Methods:**

Data including blood samples analyses and long-term clinical follow-up information for 1,261 Swedish patients with RRMS starting novel DMTs were analyzed using linear regressions to model pNfL and changes in pNfL concentrations as a function of clinical variables and DMTs (alemtuzumab, dimethyl fumarate, fingolimod, natalizumab, rituximab, and teriflunomide).

**Results:**

The baseline pNfL concentration was positively associated with relapse rate, Expanded Disability Status Scale score, Age-Related MS Severity Score, and MS Impact Score (MSIS-29), and negatively associated with Symbol Digit Modalities Test performance and the number of previously used DMTs. All analyses, which used inverse propensity score weighting to correct for differences in baseline factors at DMT start, highlighted that both the reduction in pNfL concentration from baseline to on-treatment measurement and the on-treatment pNfL level differed across DMTs. Patients starting alemtuzumab displayed the highest reduction in pNfL concentration and lowest on-treatment pNfL concentrations, while those starting teriflunomide had the smallest decrease and highest on-treatment levels, but also starting from lower values. Both on-treatment pNfL and decrease in pNfL concentrations were highly dependent on baseline concentrations.

**Conclusion:**

Choice of DMT in RRMS is significantly associated with degree of reduction in pNfL, which supports a role for pNfL as a drug response marker.

Accumulating evidence supports the notion that permanent loss of neurologic functions in multiple sclerosis (MS) is primarily correlated with the degree of damage to nerve tracts rather than degree of demyelination.^1–3^ However, due to the reserve capacity of the CNS, critical levels of nerve damage may take years to appear as clinical disability. The observation that disease-modifying therapies (DMT) used in relapsing-remitting MS (RRMS) differently affect important long-term clinical outcomes underscores a need for more sensitive measures of core disease pathologic mechanisms.^4^ MRI is the only acknowledged biomarker for disease progression and different volumetric atrophy measures have been associated with risk of developing increasing disability.^5–7^ However, such measures are insensitive to changes over shorter time periods in individual patients. Moreover, spinal cord pathology, a major driver of clinical disability, is not routinely assessed. Among different soluble markers for neuroaxonal damage, neurofilaments have emerged as promising candidates in a range of diseases.^8^ Although not specific for disease processes operating solely in MS, the potential value in this condition is especially high since it may be used to monitor treatment effects. Most published studies on neurofilament light (NfL) and effects of DMTs have measured concentrations of NfL in CSF focusing on a single or a few DMTs.^9–12^ More recently, improvements in assay sensitivity have made it possible to reliably determine NfL in serum (sNfL) or plasma (pNfL) at concentrations seen in healthy controls. Such studies have reported a correlation between baseline levels of pNfL/sNfL and measures of clinical disease activity including development of sustained disability, brain atrophy, signs of nerve tissue damage, and long-term clinical disability outcomes.^13–15^ Treatment effects have been reported by several authors.^14,16^ Disanto et al.^14^ studied 2 Swiss cohorts of patients with MS in which the effects of a limited number of DMTs on NfL were reported. In this study, the decrease in sNfL after initiation of DMT was of similar magnitude across all DMTs, but confidence intervals (CIs) were large due to the small size of the study population. Similarly, Novakova et al.^16^ reported a Swedish MS cohort in which start of DMT resulted in lowered sNfL levels, also correlating with CSF NfL concentrations, across all different DMTs, but with low power to address effect size of specific DMTs. Thus so far there is a relative paucity of well-powered studies specifically addressing treatment effects across multiple DMTs in real-world cohorts of patients. The aim of this study was to address treatment effects across multiple DMTs through the measurement of blood NfL at 2 time points in patients selected within a large cohort of patients with RRMS initiating DMT in context of a nationwide, population-based follow-up program for all newer MS DMTs.

## Methods

### Patient selection and sample collection

The Immunomodulation and Multiple Sclerosis Epidemiology study (IMSE) is a comprehensive nationwide Swedish postapproval program of patients starting newer MS DMTs, coupled with sampling of blood at initiation of therapy and at follow-up. Samples were collected from patients included in IMSE as well as in the Epidemiologic Investigation of MS and Stockholm Prospective Assessment of MS. We analyzed data for 1,139 patients with RRMS initiating alemtuzumab (ALM, n = 89), dimethyl fumarate (DMF, n = 339), fingolimod (FGL, n = 275), natalizumab (NTZ, n = 284), or teriflunomide (TFL, n = 152). Inclusion criteria comprised a baseline sample within a month prior to day of initiation of DMT and a subsequent treatment duration of >4 months. Most patients (1,052) provided 2 samples (at treatment start and on treatment [absolute range 4–24 months]). Seventeen patients (4%) contributed samples for more than 1 DMT. A follow-up program similar to IMSE was recently started for rituximab (RTX); however, only 11 of 122 analyzed patients had a sample before starting therapy. The total number of patients included in this study is thus 1,261. A total of 1,026 population-based controls included in the study by Manouchehrinia et al.^15^ was used to calculate age-adjusted pNfL reference curves.

### NfL analyses

pNfL concentrations were determined using antibodies from UmanDiagnostics (Umeå, Sweden) and the SIMOA Immunoassay using the Quanterix Kit (Quanterix, Lexington, MA). All samples from different DMTs were analyzed with blinding for treatment or clinical information. The lower limit of quantification (LLoQ) was 1.95 pg/mL. All measurements were duplicated and were above the LLoQ, with interassay and intra-assay coefficients of variation of ≤10%.

### Clinical variables collection

All IMSE patients attend regular medical visits where clinical assessments are carried out and recorded through the Swedish MS registry. In addition to general demographics (age at DMT start, age at MS onset, and sex), we had access to the dates of relapses (if any) before DMT start, the type of previous DMTs (if any) with start and stop dates and the reason for stopping, as well as clinical assessments: Expanded Disability Status Scale (EDSS), further transformed into the Age-Related MS Severity Score (ARMSS; an alternative to the Multiple Sclerosis Severity Score [MSSS] based on the patient’s age at the time of assessment^17^); the MS Impact Score (MSIS-29), divided into its physical and psychological domains; and the Symbol Digit Modalities Test (SDMT) score.

### Standard protocol approvals, registrations, and patient consents

The study was approved by the regional vetting board of Stockholm under permits 2006/845-31/1 2011/641-31/4, 2009/2017-31/2, and 04-252/1-4, with written informed consent from all participants.

### Statistical analyses

#### Variables preparation

For all analyses, we log-transformed pNfL levels to increase the normality of the distribution. We also normalized the log-pNfL values to age 40 (log-pNfL_N40_), by using the linear relationship between increasing log-pNfL and age in a large population-based control sample (i.e., log-pNfL_N40_ = log[pNfL] − 0.02115 [age at DMT start − 40]).^15^ This normalization implies that a difference between 2 pNfL_N40_ measures cannot be attributable to a difference in ages. We calculated the number of relapses in the year preceding DMT start and the number of previous DMTs (β-interferons/glatiramer acetate [IFN/GA], ALM, DMF, FGL, NTZ, RTX, and TFL) since disease onset for each patient and these 2 variables were considered as numerical. We also created a 3-category variable denoting treatment status at start of the new DMT by including a washout period (time span between stop date of previous DMT and start of new DMT) of at least 1 month for IFN/GA, DMF, and TFL, at least 3 months for FGL and NTZ, and 6 months or more for RTX (none of the patients had switched from ALM). Patients were dichotomized as being treated with IFN/GA or with one of the other DMTs if washout periods had been shorter.

#### Baseline log-pNfL levels analysis

We analyzed the log-pNfL levels at baseline (without age normalization) with linear models. Initially, we used univariable linear models with log-pNfL levels as the dependent variables and each of the variables measured at baseline (i.e., DMT start) as the independent variables to explore the correlation among log-pNfL levels, clinical variables, and patient characteristics. In a second step, we used a best subset selection approach to determine which subset of the baseline variables contributed most to explaining the variability of the pNfL levels.^18^ The tested variables included the number of previous DMTs, treatment status just before DMT start, sex, age at disease onset, disease duration, age at DMT start, number of relapses during the year before DMT start, EDSS, ARMSS, MSIS-29 (physical and psychological scales), and SDMT, all these being measured at DMT start.

#### Propensity score estimation

In order to balance the DMT groups, we calculated individual DMT propensity scores (PS), i.e., the probability to be treated with a specific DMT.^19,20^ We used a multinomial logistic model with ALM, DMF, FGL, NTZ, and TFL as the dependent variable, while the independent variables included all variables measured at DMT start, including log-pNfL_N40_. Several combinations of these variables were tested including interaction terms or transformed scales of variables. The ability of the inverse of the PS in reducing differences between DMT groups in baseline log-pNfL_N40_ values, assessed by measuring the standardized differences between the mean log-pNfL_N40_ values of each DMT group and the overall mean, depended on the input variables. Among different models tested, the one resulting in the smallest average of the standardized differences was selected.^19–21^ In the subsequent analyses, we used weights that were calculated by using the inverse of the PS. However, individual weights were limited to the 0.995th percentile of their distribution in order to prevent disproportionate effects on the analytical model.^20^ We excluded RTX from PS analyses since baseline pNfL values were available only for a small minority.

#### Changes in log-pNfL_N40_ levels analysis

We used a graphical approach to describe changes in log-pNfL_N40_ levels from DMT start to follow up (4–24 months later) using unweighted means of the log-pNfL_N40_ across different DMTs, and subsequently, values weighted by the inverse of the PS. As the main question was to assess if different DMTs were significantly associated with degree of reduction of pNfL_N40_ concentrations, we calculated the delta pNfL_N40_ (i.e., change in log-pNfL_N40_ levels). We used a weighted linear model with delta as the dependent variable and the DMTs as the independent variable, using weights obtained by inversing the PS, and further adjusted for other baseline covariates to remove potential residual confounding.^22^ Criteria to retain a variable included percentage of the explained variance, and how much the additional variable modified the estimates for the DMTs. In an additional sensitivity analysis, we stratified on the quintiles of the PS instead of using weights. We also analyzed how the changes in log-pNfL_N40_ correlated with the changes in EDSS, ARMSS, MSIS-29, and SDMT using univariable models.

#### Additional supporting analyses

As RTX was excluded from the analyses using PS, we also modeled the log-pNfL_N40_ on treatment, without using PS but adjusting the analyses for patient characteristics using linear models. In parallel, the log-pNfL_N40_ on treatment without RTX but using PS was also modeled.

### Data availability

Data related to the current article are available from Tomas Olsson, Karolinska Institutet. To share data from the Swedish MS registry, a data transfer agreement needs to be completed between Karolinska Institutet and the institution requesting data access. This is in accordance with data protection legislation in Europe (General Data Protection Regulation). Persons interested in obtaining access to the data should contact Tomas Olsson at tomas.olsson@ki.se.

## Results

### Baseline characteristics

**Figure 1:**
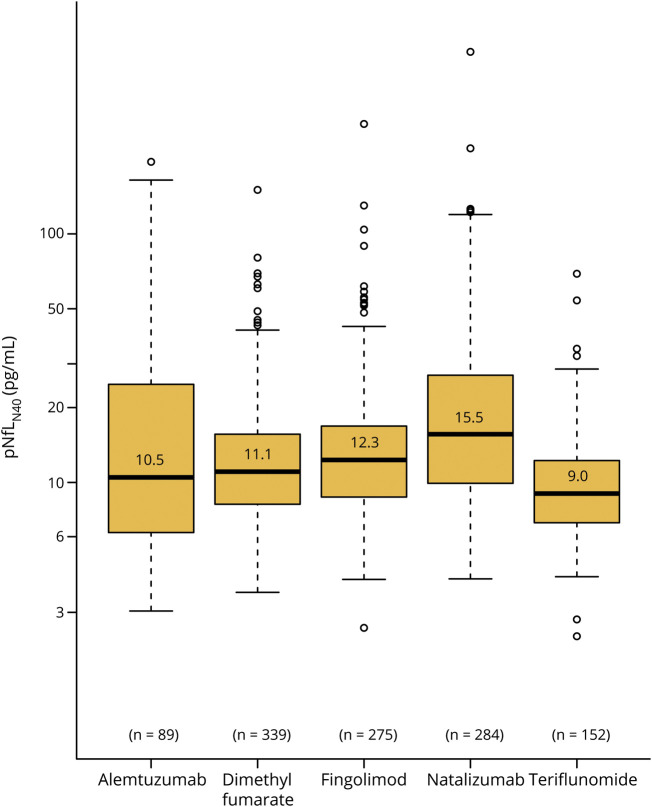
Baseline log–neurofilament light in plasma (pNfL)_N40_ levels in groups starting different disease-modifying therapies (DMTs) (with median and 25th and 75th percentiles) Box and whisker plots show the distributions of the log-pNfL_N40_ concentrations in each group of patients at DMT start.

Data on baseline patient characteristics at therapy initiation are presented in table 1. There were large differences between DMT groups, where for example those starting TFL were older both at disease onset and at therapy initiation, had lower MSIS-29 and ARMSS values, and had a longer disease duration compared to other DMT groups (table 1). From a disease severity perspective, NTZ starters were characterized by both higher EDSS and MSIS-29 scores as well as higher relapse activity compared to other groups. These differences were mirrored in both baseline pNfL (data not shown) and baseline pNfL_N40_ concentrations (table 1 and figure 1).

**Table 1:**
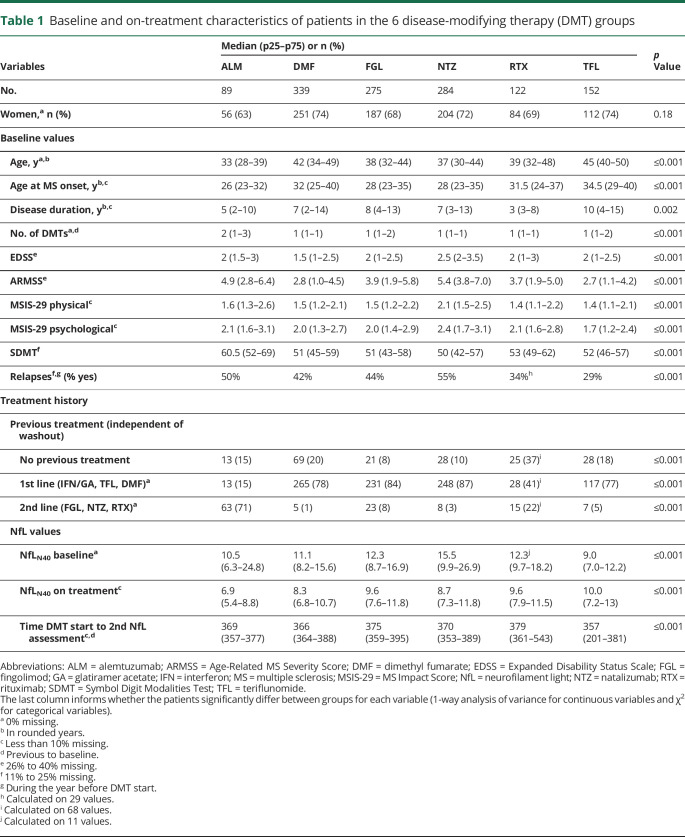
Baseline and on-treatment characteristics of patients in the 6 disease-modifying therapy (DMT) groups

### Modeling baseline log pNfL

The pNfL values displayed a skewed distribution and were log transformed. We then modeled log-pNfL levels at baseline (without age normalization) with a linear model. As most of the variables displayed a fluctuating degree of association with the pNfL values and also interacted, we used a best subset selection to model pNfL variability across groups. The back transformed estimates (exp[β]) are given in table 2 for both the univariable and multivariable models. The pNfL levels increased with EDSS, ARMSS, MSIS-29 (physical and psychological scales), and number of relapses before DMT start, and decreased with SDMT scores and number of previous DMTs.

**Table 2:**
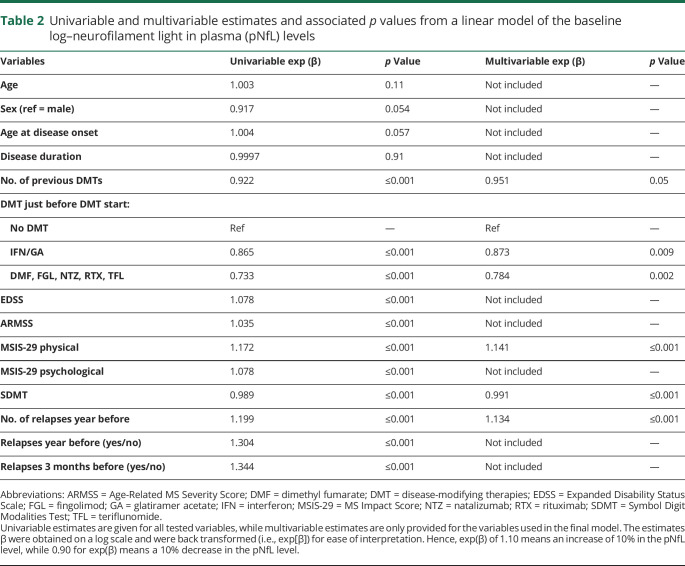
Univariable and multivariable estimates and associated *p* values from a linear model of the baseline log–neurofilament light in plasma (pNfL) levels

### Propensity scores

**Figure 2:**
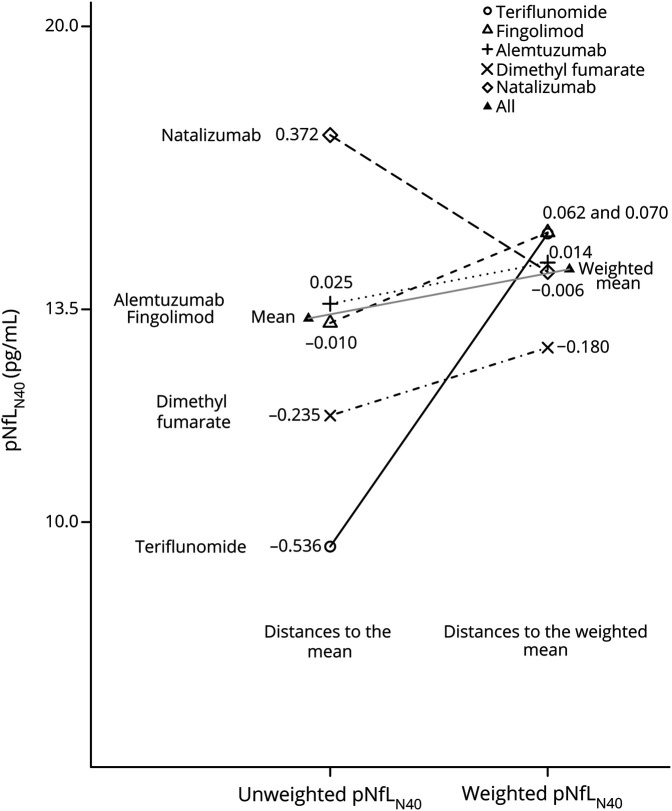
Unweighted and weighted baseline log–neurofilament light in plasma (pNfL)_N40_ The ability of the propensity scores to correct the imbalance between disease-modifying therapy groups is shown graphically and numerically for log-pNfL_N40_ levels. The distances are standardized (i.e., they do not depend on the unit in which the variable was measured). The effect of the propensity score is to decrease the standardized distances, where a standardized distance larger than 0.20 can be considered as evidence of imbalance and a potential source of bias.^21^ Here, there is some small residual imbalance for dimethyl fumarate. The average of the absolute standardized distances was 0.24 before weighting and 0.05 after weighting.

The variables retained for modeling the PS through the multinomial logistic model of the 5 DMTs, excluding RTX, were selected after testing several combinations of the baseline variables, retaining the model with the smallest average standardized difference. This model included the baseline pNfL_N40_ level, ARMSS, EDSS, SDMT, age at disease onset, the number of previous DMTs, the treatment status just before starting the new DMT, and the number of relapses during the year before DMT start. With these variables, the average of the standardized absolute distances for log-pNfL_N40_ dropped from 0.24 before weighting to 0.05 after weighting (figure 2).

### Changes in log-pNfL_N40_ levels analysis

**Figure 3:**
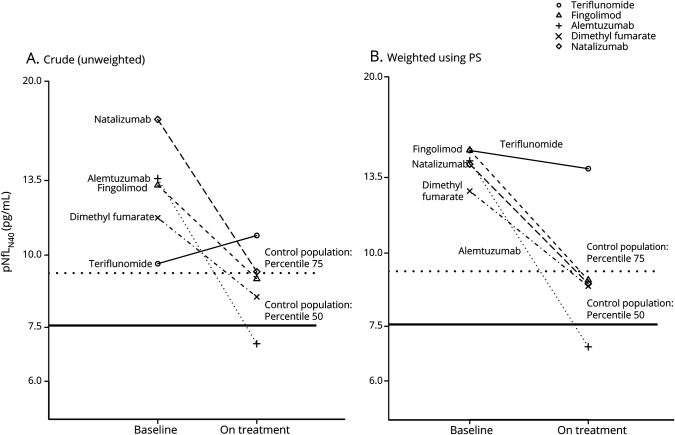
Baseline and on-treatment mean neurofilament light in plasma (pNfL)_N40_ levels in the disease-modifying therapy groups (A) Crude mean pNfL levels at baseline and on treatment. (B) Weighted mean pNfL levels at baseline and on treatment. The weights are the inverse of the propensity scores.

The changes in log-pNfL_N40_ levels between baseline and on treatment are presented in figure 3, both for the unweighted values (figure 3A) and the values weighted with the inverse of the PS (figure 3B). Despite PS weighting, some differences between DMTs remained, suggesting residual effects of factors not accounted for. The estimates from both the unweighted and weighted linear regression models with delta (i.e., change in log-pNfL_N40_) as the dependent variable and the DMTs as explanatory variables are presented in table 3. The estimates (β) were back-transformed to the original scale (exp[β]), so that, for example, a value of 0.80 translates into a 20% reduction of the baseline pNfL_N40_ level. The mean change was affected by the type of DMT, with the largest mean reduction for ALM in both analyses (54% reduction [95% CI 43%–62% reduction] and 48% reduction [49%–56% reduction] respectively for the unweighted and weighted analyses) and the smallest change for TFL, for which the significance level of 0.05 was not reached (12% increase [3% reduction to 29% increase] and 7% reduction [16% reduction to 4% increase] respectively for the unweighted and weighted analyses). A post hoc analysis highlighted similarities and differences between DMT groups; the mean delta between DMF and FGL and between NTZ and ALM were not statistically different for the unweighted model. In the weighted model, the mean delta of NTZ did not differ significantly from DMF and FGL (data not shown). To remove any residual confounding, we further adjusted our model with several baseline covariates. While this dramatically increased the percentage of the variance explained, it did not change the pattern observed with our first (weighted) model. The estimates were only slightly modified when including the log-pNfL_N40_ at baseline in the model (table 4). Similar limited changes also occurred with inclusion of additional baseline covariates or stratification on PS quintiles (instead of weighting) (table 4). In order to explore the effect of previous DMTs, we further stratified on previous treatment and on baseline pNfL_N40_ level (data not shown). This provided additional insights without modifying our previous observations. Finally, we also observed that the changes in log-pNfL_N40_ values, EDSS, ARMSS, and MSIS-29 were all significantly correlated to each other, though often with low correlation coefficients (i.e., around 0.3 or below).

**Table 3:**
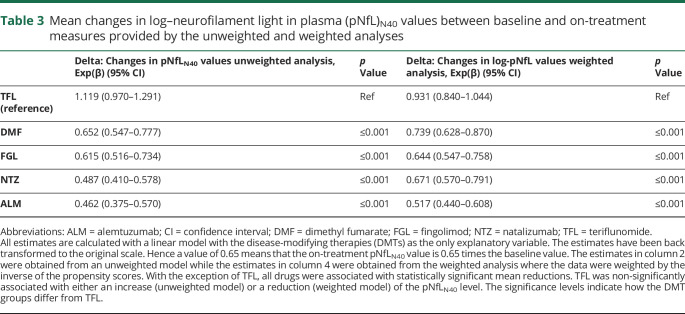
Mean changes in log–neurofilament light in plasma (pNfL)_N40_ values between baseline and on-treatment measures provided by the unweighted and weighted analyses

**Table 4:**
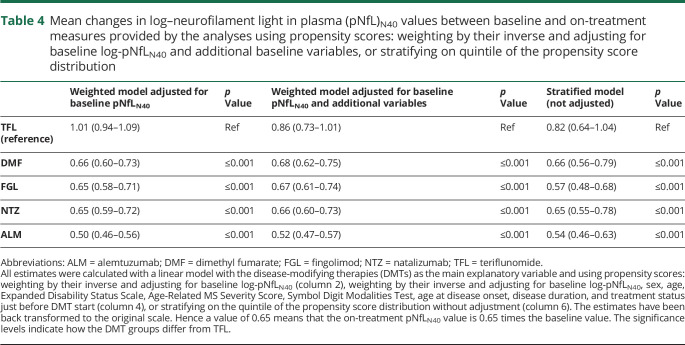
Mean changes in log–neurofilament light in plasma (pNfL)_N40_ values between baseline and on-treatment measures provided by the analyses using propensity scores: weighting by their inverse and adjusting for baseline log-pNfL_N40_ and additional baseline variables, or stratifying on quintile of the propensity score distribution

### On-treatment log-pNfL_N40_ levels

The analysis of the log-pNfL_N40_ on treatment with either a weighted linear model (without RTX group) or with an unweighted model showed that all DMT groups had on average lower values than TFL (table 5). Adjusting for the baseline log-pNfL_N40_ improved the model substantially, increasing the percentage of the explained variance from 21% to 40%, but did not affect overall estimates. Additional adjustments did not substantially modify these estimates further. Treatment duration was tested but did not have a significant contribution.

**Table 5:**
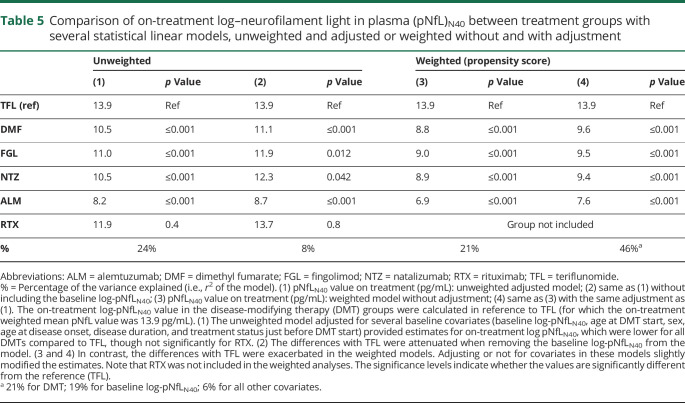
Comparison of on-treatment log–neurofilament light in plasma (pNfL)_N40_ between treatment groups with several statistical linear models, unweighted and adjusted or weighted without and with adjustment

## Discussion

Disease pathogenesis in RRMS evolves over years and the availability of a growing number of treatment options creates a need for additional means to assess disease activity and response to treatment, including body fluid biomarkers.^8,23^ In addition, real-world studies conducted in unselected patient populations can provide important information on questions that cannot be addressed with existing data from randomized controlled trials.^24^ Along these lines, we explored how pNfL concentrations were distributed in patients with RRMS starting newer DMTs, how this distribution was associated with clinical measures and patient characteristics, and how pNfL concentrations evolved under treatment. Strengths of the study include the possibility to simultaneously compare across multiple treatments in nonrestricted patient groups, but this approach also entails major challenges in balancing out differences in baseline characteristics, since DMT selection is heavily influenced by clinical disease characteristics. Nevertheless, by modeling on relevant variables, we demonstrate that the reduction in pNfL concentrations differs across DMTs, with the largest reduction for ALM and the smallest for TFL. This result is largely in agreement with the perceived effectiveness of the studied DMTs. Still, reductions in pNfL with DMF, FGL, and NTZ were similar even if NTZ generally is considered to have a superior effect on relapses and focal MRI lesions of the 3. This observation may be partly explained by indication bias (i.e., patients with more active disease are started on highly effective drugs); however, an interesting feature with pNfL is that it reflects both diffuse and focal neuroaxonal damage, where it may be speculated whether different DMTs affect these 2 aspects differently, for example based on their capacity to penetrate into the CNS. This will need longer follow-up studies that also integrate quantitative MRI measures. Also, the kinetics of how pNfL is affected might differ across DMTs, necessitating longer follow-up with repeated sampling. Finally, comorbidities affecting the peripheral nervous system or CNS may act as confounders. For example, leflunomide, which is related to TFL, has been shown to affect the peripheral nervous system.^25^ An additional important finding is that we show how essential the baseline pNfL concentration is for correctly predicting the pNfL concentration on treatment. In fact, the percentage of the variance explained by the baseline concentration (>20%) outsized all other factors. Accordingly, inclusion of the baseline pNfL value affected estimates, increasing the differences in pNfL concentrations between the DMTs. We also find that reductions in pNfL concentrations correlated with improvements in clinical variables, such as EDSS, MSSS, and MSIS-29, though correlation coefficients were low (between 0.10 and 0.30), replicating earlier findings.^13,14^ Importantly, as shown by recent studies, pNfL concentrations at diagnosis also predict important long-term outcomes, such as brain atrophy and risk to achieve clinical disability milestones.^15,26^

Whereas our data reveal differences in pNfL dynamics across the studied DMTs, we cannot rule out that differences had been achieved with a more complete model for the PS, even if our additional adjustments did not lead to major changes in the estimates. Notably, however, we did not have access to sufficiently precise MRI data, which are known to affect pNfL.^13^ A further weakness is imprecise information on some measures, e.g., the lack of coding for switching from NTZ due to positive JC virus serology in the Swedish MS registry. On the other hand, the high general validity of data entered into the Swedish MS registry regarding treatment episodes and relapses was recently confirmed by a large-scale national validation against medical records.^27^ Furthermore, most patients in the RTX group lacked a baseline sample, which meant that this group was excluded from analyses involving PS and that other analyses including baseline log-pNfL_N40_ became less precise. Also the proportion of patients missing information for some variables that were used in the adjustment (or in the PS estimation) could also have hampered the power of our study. The observational design of the study implies that patients were not randomized to treatment, nor were they randomly selected within the IMSE cohorts, and therefore some selection bias could have occurred. It is therefore important to relate these findings to studies exploring pNfL concentrations in the context of randomized control trials, even if such studies rarely include more than 2 DMTs.^28^ As a final note, the extent different DMTs affected pNfL largely mimic their effect on the long-term risk to convert to a secondary progressive disease course, as observed in a large recent real-world study.^4^ The implementation of soluble but also novel imaging biomarkers that can complement current clinical and imaging monitoring likely will lead to an increased use of more effective DMTs and reduce the risks for patients to be exposed to insufficient treatment responses, in turn improving important long-term clinical outcomes.^12,29^

We demonstrate that dynamics of pNfL are significantly influenced by specific DMTs and that the degree of pNfL reduction is correlated to clinical and patient-reported outcomes, but also that the baseline pNfL concentration exerts an unproportioned effect on on-treatment values in the medium term. In order to understand if pNfL can be used as a drug response biomarker at the individual level, further studies are needed to address the correlation of pNfL changes to long-term clinical outcomes with different DMTs, as well as if modeling of pNfL dynamics can be improved further by including additional variables such as MRI data or more frequent measurements.

## Glossary

ALM: alemtuzumab
ARMSS: Age-Related MS Severity Score
CI: confidence interval
DMF: dimethyl fumarate
DMT: disease-modifying therapies
EDSS: Expanded Disability Status Scale
FGL: fingolimod
GA: glatiramer acetate
IFN: interferon
IMSE: Immunomodulation and Multiple Sclerosis Epidemiology
LLoQ: lower limit of quantification
MS: multiple sclerosis
MSIS-29: MS Impact Score
MSSS: Multiple Sclerosis Severity Score
NfL: neurofilament light
NTZ: natalizumab
pNfL: neurofilament light in plasma
PS: propensity scores
RRMS: relapsing-remitting multiple sclerosis
RTX: rituximab
SDMT: Symbol Digit Modalities Test
sNfL: neurofilament light in serum
TFL: teriflunomide
